# Comparison of *in vitro* antimicrobial susceptibility between mucoid and non-mucoid *Pseudomonas aeruginosa* and its guiding value for antibiotic therapy

**DOI:** 10.1128/spectrum.00287-25

**Published:** 2025-06-11

**Authors:** Yun Wu, Aixin Wang, Jinghan Qu, Ruirui Ma, Wei Kang, Yali Liu, Yingchun Xu

**Affiliations:** 1 Department of Laboratory Medicine, State Key Laboratory of Complex Severe and Rare Diseases, Peking Union Medical College Hospital, Chinese Academy of Medical Sciences and Peking Union Medical College 12501, Beijing, China; 2 Graduate School, Peking Union Medical College, Chinese Academy of Medical Sciences 12501, Beijing, China; 3 Department of Pharmacy, Peking Union Medical College Hospital, Chinese Academy of Medical Sciences and Peking Union Medical College 12501, Beijing, China; University of Debrecen, Debrecen, Hungary

**Keywords:** *Pseudomonas aeruginosa*, mucoid, clinical characteristic, antimicrobial susceptibility, antimicrobial agents

## Abstract

The aim of the study was to compare *in vitro* antimicrobial susceptibility between mucoid and non-mucoid *Pseudomonas aeruginosa* and its guiding value for antibiotic therapy. A retrospective review was conducted on the sources of isolates, patient information, *in vitro* antimicrobial susceptibility results, and antimicrobial usage records for mucoid and non-mucoid *P. aeruginosa* isolates spanning from 2021 to 2023, comparing the differences between the two types. Additionally, infection cases of mucoid and non-mucoid *P. aeruginosa* were selected for in-depth analysis of their treatment courses, evaluating the guiding value of antimicrobial susceptibility results for antimicrobial therapy. We collected 1,443 non-mucoid *P. aeruginosa* and 342 mucoid *P. aeruginosa*. Our findings revealed that mucoid *P. aeruginosa* was predominantly found in respiratory tract specimens, while non-mucoid *P. aeruginosa* was primarily isolated from a broader range of specimen sources. Regarding antimicrobial susceptibility, mucoid *P. aeruginosa* exhibited notably lower levels of resistance compared to their non-mucoid counterparts, except for ciprofloxacin, levofloxacin, and cefepime. The most frequently prescribed drugs for mucoid *P. aeruginosa* were levofloxacin and ceftazidime, whereas for non-mucoid *P. aeruginosa,* ceftazidime and meropenem were more frequently used. A total of 42 non-mucoid *P. aeruginosa* cases and 36 mucoid *P. aeruginosa* cases were further analyzed, which were isolated from hospitalized patients with a confirmed diagnosis of monomicrobial *P. aeruginosa* infection. The analysis of case information revealed that 90.2% of non-mucoid *P. aeruginosa* infections and 94.4% of mucoid *P. aeruginosa* infections showed consistency between *in vitro* antimicrobial susceptibility and outcomes. Our results revealed differences in demographic and clinical characteristics, antimicrobial susceptibility, and selection of antimicrobial agents between mucoid and non-mucoid *P. aeruginosa*. The findings will help optimize prognosis and combat the challenges posed by *P. aeruginosa* infections.

**IMPORTANCE:**

This study represents a large-scale comparative research conducted over two years at a tertiary hospital in China, involving 1,443 non-mucoid and 342 mucoid strains. We found significant differences between mucoid and non-mucoid *P*. *aeruginosa* infections in terms of gender, antimicrobial susceptibility test, and the selection of antimicrobial agents. Specifically, mucoid *P. aeruginosa* infections were predominantly observed in females and were mainly associated with respiratory tract infections. Moreover, the isolates with mucoid phenotype exhibited higher susceptibility to most antimicrobial agents compared to non-mucoid isolates. According to the data of the pharmacy department, we found the most frequently prescribed drugs for mucoid *P. aeruginosa* were levofloxacin and ceftazidime, whereas for non-mucoid *P. aeruginosa*, ceftazidime and meropenem were more frequently used. The analysis of case information revealed that no matter whether in mucoid or non-mucoid *P. aeruginosa*, the in vitro antimicrobial susceptibility test results were in good agreement with the therapeutic outcomes.

## INTRODUCTION

*Pseudomonas aeruginosa* is an opportunistic pathogen, posing serious infection risks to patients undergoing mechanical ventilation, individuals who are immunocompromised, and those with malignancies or HIV infection. Hospital-acquired infections are common, and chronic airway infections with *P. aeruginosa* are a significant co-morbidity in patients with cystic fibrosis (CF), bronchiectasis, chronic obstructive pulmonary disease (COPD), or ventilator-associated pneumonia (VAP) (1–3).

*P. aeruginosa* can be classified into mucoid and non-mucoid types based on colony morphology. Mucoid *P. aeruginosa* displays a slower growth rate, forming smaller colonies when cultured at 35°C for 24 hours, which can lead to their easy oversight (4). Previous studies have confirmed differences in antimicrobial susceptibility (3, 5), as well as in treatment and prognosis (6), between mucoid and non-mucoid *P. aeruginosa*. Considering the difficulty in performing *in vitro* antimicrobial susceptibility testing for *P. aeruginosa* (7), especially for mucoid isolates, it is uncertain whether *in vitro* susceptibility to antimicrobial agents can accurately guide the selection of antimicrobial agents. Foreign studies indicated that mucoid *P. aeruginosa* is predominantly isolated from cystic fibrosis patients (8–10); however, cystic fibrosis patients are rare in China. The main diseases caused by mucoid *P. aeruginosa* in China are still unclear. Additionally, some research has highlighted differences in disease progression and outcomes between mucoid and non-mucoid *P. aeruginosa* infections (11). Therefore, it’s essential to conduct a comparative study between mucoid and non-mucoid *P. aeruginosa*, focusing on demographic and clinical characteristics, *in vitro* antimicrobial susceptibility, as well as the selection of antimicrobial agents. Moreover, it’s vital to evaluate the guiding value of *in vitro* antimicrobial susceptibility testing for antimicrobial therapy.

This study examined the specimen sources, gender distribution, age groups, clinical diagnosis, and antimicrobial susceptibility of 1,443 non-mucoid *P. aeruginosa* and 342 mucoid *P. aeruginosa* isolated from a tertiary hospital in China, Peking Union Medical College Hospital, between 2021 and 2023. We also analyzed the usage of antimicrobial agents among mucoid and non-mucoid *P. aeruginosa*, comparing differences between outpatients and inpatients, as well as before versus after antimicrobial susceptibility reports. Additionally, we also evaluated the consistency between the results of antimicrobial susceptibility *in vitro* and the treatment outcome. The primary aim of this analysis was to provide robust insights for the clinical prevention and treatment of *P. aeruginosa* infections.

## MATERIALS AND METHODS

### *P. aeruginosa* isolates and clinical data collection

This study focused on 1,443 non-mucoid *P. aeruginosa* and 342 mucoid *P. aeruginosa*, isolated from a diverse range of clinically submitted and qualified specimens at Peking Union Medical College Hospital between 2021 and 2023. Only the first isolate from the same site in the same patient was included. To analyze the differences in the usage of antimicrobial agents between mucoid and non-mucoid *P. aeruginosa* infection, we retrieved medication usage data from the Hospital Information System for the treatment of *P. aeruginosa*. Clinical information, including patient’s gender, age, isolated department, and infection site, was also obtained from the Hospital Information System.

### Bacterial identification and Antimicrobial Susceptibility Test

All isolates were positive in the oxidase test and identified as *P. aeruginosa* through matrix-assisted laser desorption/ionization time-of-flight (MALDI-TOF) mass spectrometry (Smart MS, Zhuhai DL Biotech Co., Ltd). Antimicrobial susceptibility test (AST) of mucoid *P. aeruginosa* was performed using the disk diffusion method. For non-mucoid *P. aeruginosa,* AST was carried out using VITEK2 N335 (gram-negative bacteria susceptibility test card; BioMérieux) and the disk diffusion method. Interpretation of the AST results was based on the clinical breakpoints of M100-Ed34 of the Clinical and Laboratory Standards Institute (CLSI) 2024 (12). *Escherichia coli* (ATCC 25922) and *P. aeruginosa* (ATCC 27853) were used as quality controls.

### Correlation of *in vitro* antimicrobial susceptibility and clinical outcome

Among the 1,143 non-mucoid *P. aeruginosa* and 342 mucoid *P. aeruginosa* with antimicrobial susceptibility data, 56 non-mucoid isolates and 57 mucoid isolates, which were isolated from hospitalized patients with a confirmed diagnosis of monomicrobial *P. aeruginosa* infection, were selected for case information retrieval. Cases with incomplete information or those without antibiotic treatment were excluded. Ultimately, 42 non-mucoid *P. aeruginosa* cases and 36 mucoid *P. aeruginosa* cases were involved in our study. Concordance between *in vitro* antimicrobial susceptibility testing and clinical outcomes was considered if the antimicrobial agents used in the treatment were susceptible *in vitro* and the treatment outcome was either cured or improved, or if the antimicrobial agents were resistant *in vitro* and the outcome was either not cured or resulted in death. Any other discrepancies were classified as discordant. Detailed sample data are shown in Fig. S1.

### Statistical approach

All statistical analyses were performed using Statistical Package for the Social Sciences version 29.0 software (SPSS Inc., Chicago, IL, USA). Descriptive analysis was conducted, and differences in specimen sources, gender distribution, age groups, clinical distribution, and antibiotic resistance rate between mucoid and non-mucoid isolates were assessed using Pearson’s *χ^2^* test or correction for continuity. A *P* value of < 0.05 was considered statistically significant.

## RESULTS

### Isolates and patients information

**Fig 1:**
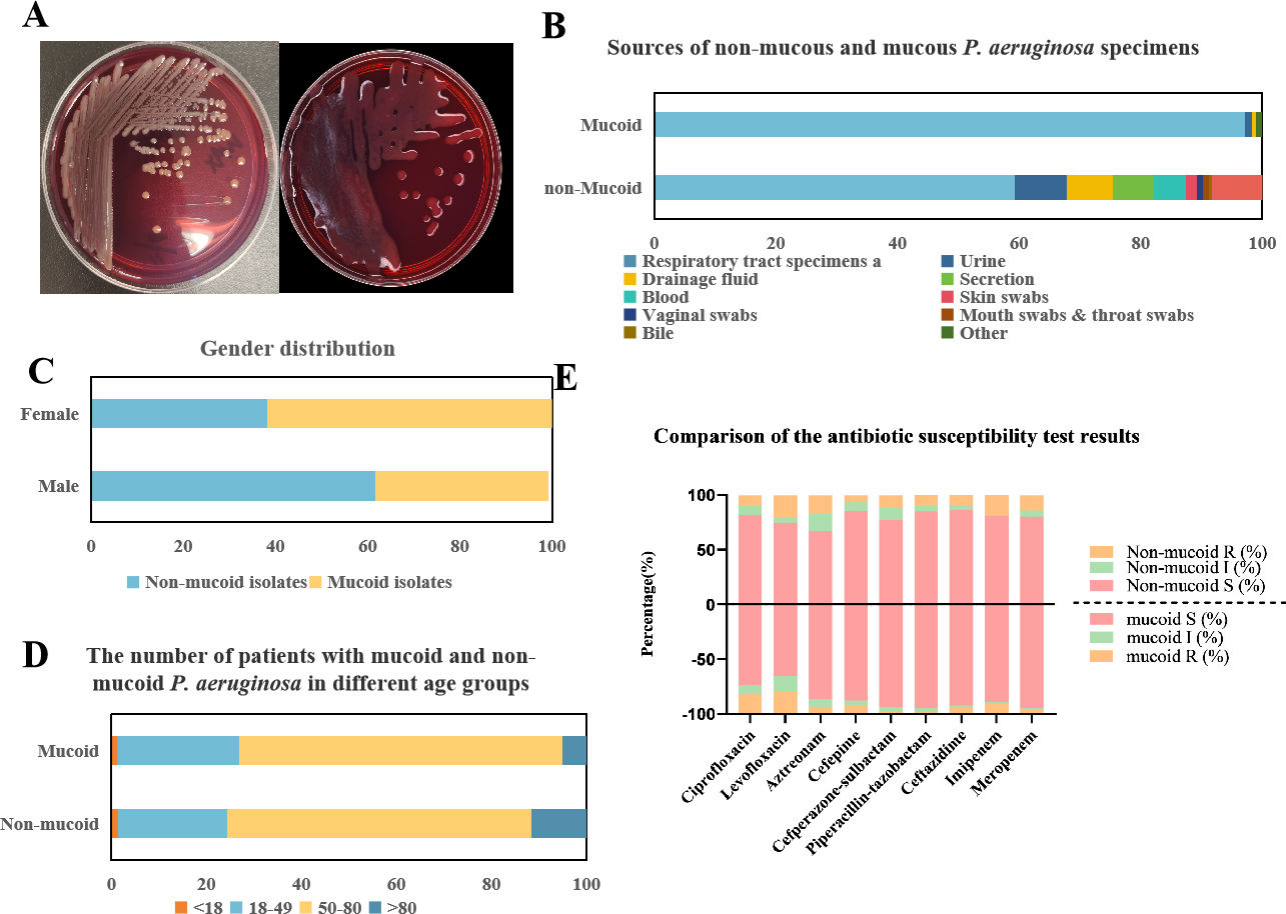
Clinical information of the collected isolates. (A) Comparison of mucoid and non-mucoid *P. aeruginosa* colonies (cultured at 35°C for 24 hours on CBA). Left: the colony morphology of non-mucoid *P. aeruginosa*. Right: the colony morphology of mucoid *P. aeruginosa*. (B) Gender distribution of *P. aeruginosa* mucoid and non-mucoid type. (C) The number of patients with mucoid and non-mucoid *P. aeruginosa* in different age groups. (**D**) Sources of non-mucoid and mucoid *P. aeruginosa* specimens. Respiratory tract specimens include sputum, tracheobronchial aspirate, endotracheal aspirate, and bronchoalveolar lavage fluid. (E) Comparison of the antimicrobial susceptibility test results for non-mucoid and mucoid *P. aeruginosa* to different antibiotics. Left: Mucoid isolates; Right: Non-mucoid isolates.

We collected 1,443 non-mucoid and 342 mucoid *P. aeruginosa*. The sources of the specimens for both mucoid and non-mucoid *P. aeruginosa* are illustrated in Fig. 1B. Mucoid *P. aeruginosa* isolates were predominantly isolated from respiratory specimens, accounting for 97% of the total. In contrast, non-mucoid *P. aeruginosa* isolates isolated from respiratory specimens comprised 45% of the total (*P* < 0.001), with 8.5% originating from urine and 7.5% from drainage fluid.

Notably, a statistically significant gender disparity was observed (*P* < 0.001), with mucoid *P. aeruginosa* isolates being predominantly associated with female patients (62.6%, 214/342), while non-mucoid isolates were predominantly found in male patients (61.7%, 890/1,443). The distribution of patients with mucoid and non-mucoid *P. aeruginosa* across different age groups is shown in Fig. 1D. A statistically significant difference was observed in the percentage of isolates for individuals above 80 years (*P* < 0.001).

### Comparison of antimicrobial susceptibility results

Figure 1E presented a comparison of antimicrobial susceptibility results for non-mucoid and mucoid *P. aeruginosa* across nine commonly used antimicrobial agents. Among the nine different antimicrobial agents, it was evident that mucoid *P. aeruginosa* generally exhibited notably lower resistance levels in comparison to non-mucoid isolates (*P* < 0.001), except for ciprofloxacin and cefepime. Specifically, mucoid *P. aeruginosa* exhibited significantly higher resistance to ciprofloxacin compared to non-mucoid isolates (*P* < 0.001). The lowest resistance rate observed among mucoid isolates was piperacillin-tazobactam (1.7%), while non-mucoid isolates exhibited the lowest resistance rate to cefepime (6.8%). Both mucoid and non-mucoid *P. aeruginosa* isolates demonstrated the highest resistance rates against levofloxacin, exceeding 20%.

### Selection of antimicrobial agents in mucoid and non-mucoid *P. aeruginosa*

**Fig 2:**
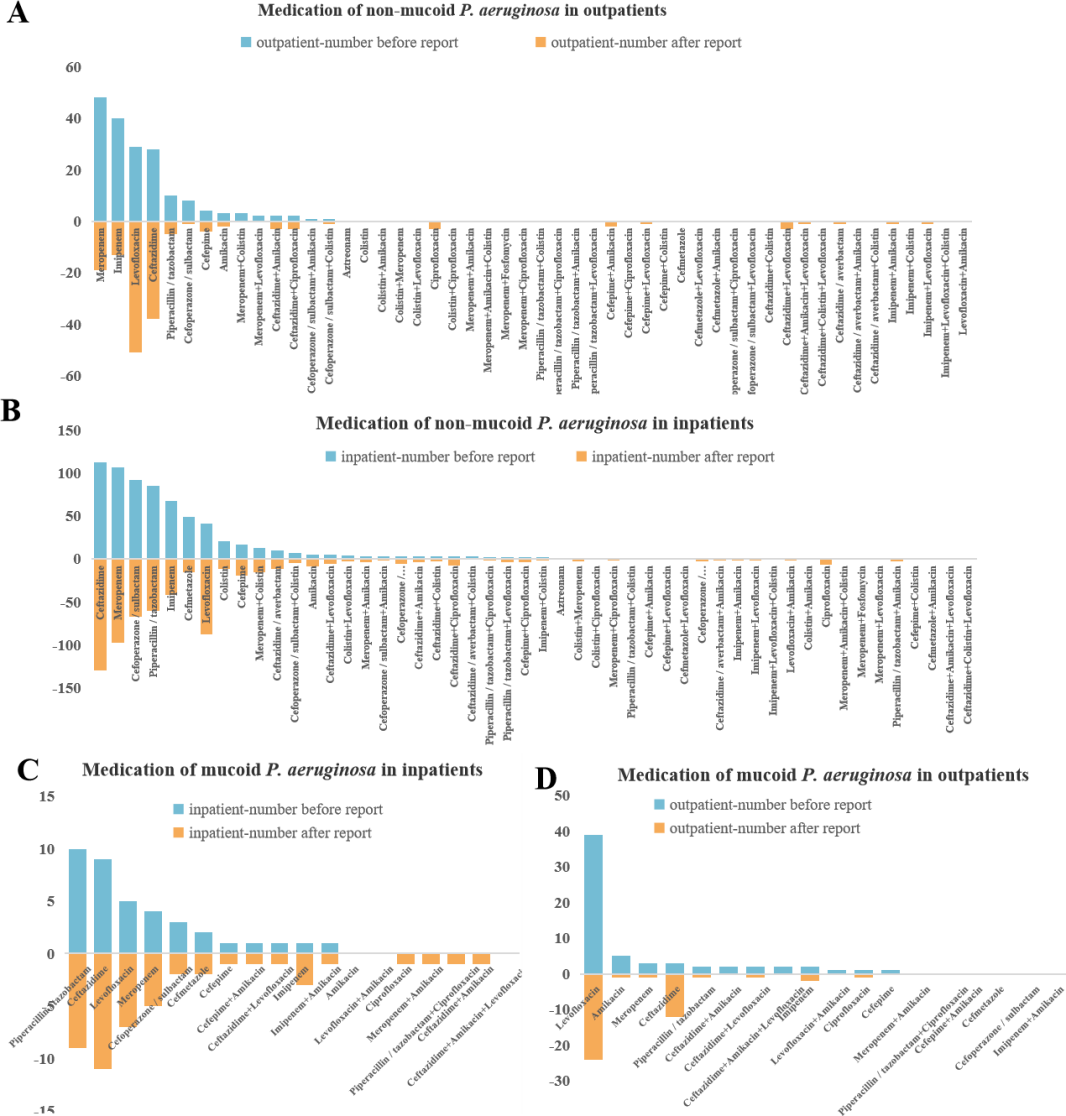
Medication of mucoid and non-mucoid *P. aeruginosa*. A through D.

Figure 2 shows the medication use profiles of mucoid and non-mucoid *P. aeruginosa* in both outpatient and inpatient departments. A notable difference between the two groups was observed in the medication use. There are seven kinds of monotherapy and nine kinds of combined therapy for mucoid *P. aeruginosa,* while for non-mucoid *P. aeruginosa*, there are eight kinds of monotherapy and 39 kinds of combined therapy. We observed specific antimicrobial agent preferences in the treatment of mucoid and non-mucoid *P. aeruginosa*. In mucoid *P. aeruginosa*, the most frequently prescribed antimicrobial agent was levofloxacin, followed by ceftazidime and piperacillin/tazobactam. Treatment preferences differed between outpatients and inpatients with mucoid *P. aeruginosa*; piperacillin/tazobactam was most commonly used in hospitalized patients (Fig. 2C), whereas levofloxacin was preferred for outpatients (Fig. 2D). For non-mucoid *P. aeruginosa*, ceftazidime and meropenem were the most commonly prescribed antimicrobial agents overall. In hospitalized patients, ceftazidime was most frequently used (Fig. 2B), followed by meropenem, whereas levofloxacin was the more popular antimicrobial agent choice for outpatients (Fig. 2A).

A definitive antimicrobial susceptibility report significantly influences medication decisions. Among outpatients, 9.78% (9/92) of antimicrobial agents for mucoid *P. aeruginosa* were adjusted following the microbiological report, compared to 11.50% (32/287) for non-mucoid *P. aeruginosa* (Fig. 2A, Fig. 2D). For hospitalized patients, 22.91% (11/48) of mucoid *P. aeruginosa* treatments and 18.10% (148/817) of non-mucoid *P. aeruginosa* treatments were adjusted post-report (Fig. 2B, Fig. 2C). Compared with the situation that clinicians administer drugs without knowing a definitive antimicrobial susceptibility report, after the report, there was an increase in the use frequency of ceftazidime and levofloxacin, while the use frequency of meropenem and piperacillin/tazobactam decreased in non-mucoid *P. aeruginosa*. The antimicrobial agents adjustment of mucoid *P. aeruginosa* was similar to non-mucoid *P. aeruginosa,* except for mucoid *P. aeruginosa* in outpatients, where the frequency of levofloxacin decreased. After the report, in both mucoid and non-mucoid *P. aeruginosa,* the usage of ciprofloxacin became more frequent.

### Correlation of *in vitro* antimicrobial susceptibility and clinical outcome

A total of 42 non-mucoid *P. aeruginosa* cases and 36 mucoid *P. aeruginosa* cases were included with complete case information. The analysis results of case information are shown in Table S1. The most commonly used antimicrobial agents for non-mucoid *P. aeruginosa* were meropenem (15/42, 35.7%), followed by ceftazidime (12/42, 26.1%). For mucoid *P. aeruginosa*, the most commonly used antimicrobial agent was ceftazidime (12/36, 33.3%). Among non-mucoid *P. aeruginosa* cases, 78.6% were treated with combined therapy, while only 54.1% of mucoid *P. aeruginosa* cases were treated with combined therapy. Additionally, we analyzed the consistency between the antimicrobial susceptibility results and the outcomes (improvement or no cure) for the antimicrobial agents used. The results showed that 90.2% of non-mucoid *P. aeruginosa* infections and 94.4% of mucoid *P. aeruginosa* infections showed consistency between *in vitro* antimicrobial susceptibility and outcomes. In the case of mucoid and non-mucoid *P. aeruginosa* infection, there were two and three cases, respectively, where the *in vitro* antimicrobial susceptibility was sensitive, yet the clinical treatment outcome was either death or failure to cure. Among infections caused by non-mucoid *P. aeruginosa*, there were two cases where the *in vitro* antimicrobial agents were resistant, but the clinical outcome was improved; no such cases were observed in infections caused by mucoid *P. aeruginosa*.

## DISCUSSION

**Fig 3:**
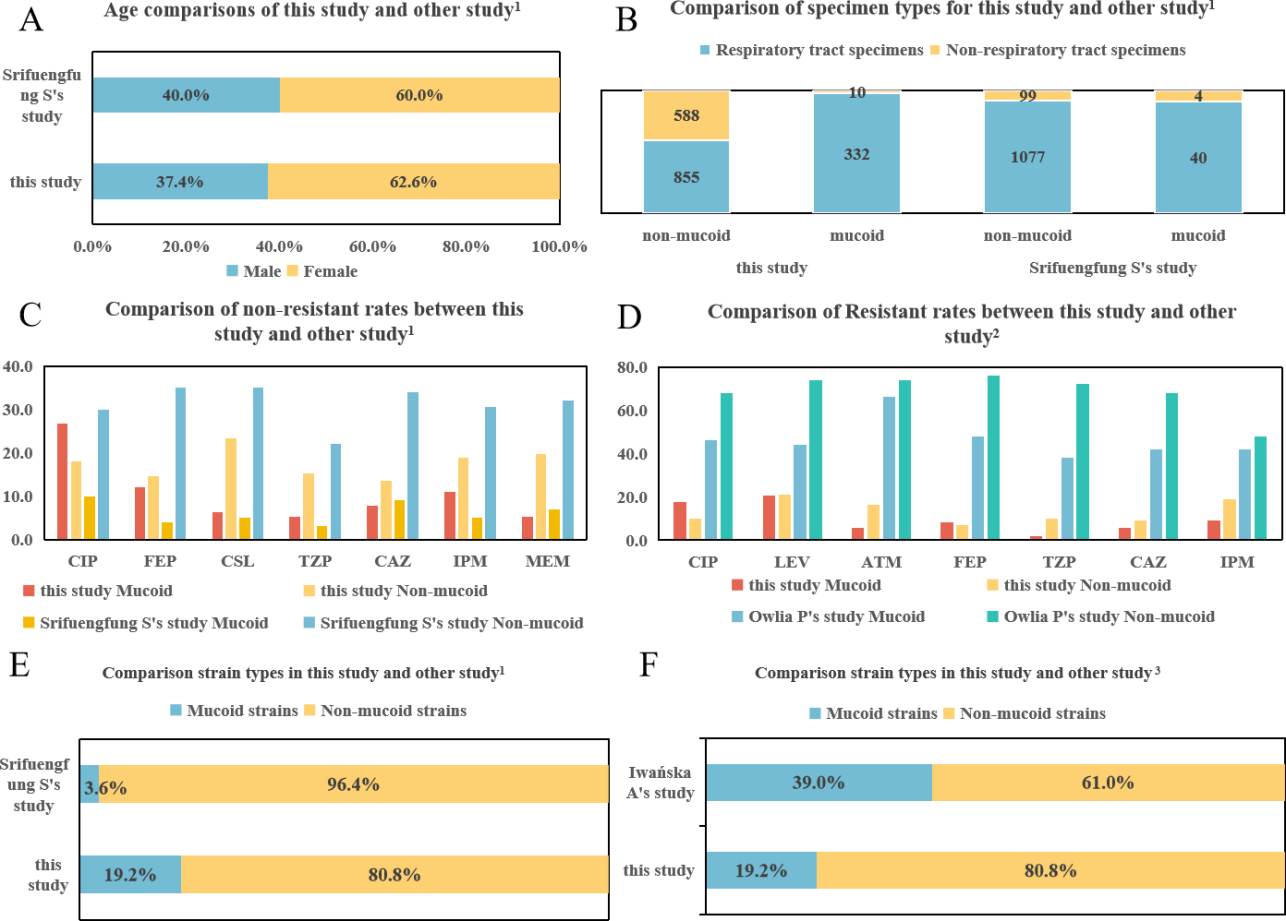
Comparison of home and foreign data. *Reference in this figure. (3, 5, 14). A through F.

*P. aeruginosa* can be categorized into mucoid and non-mucoid types based on their colony morphology, and in the infection caused by different morphotypes of *P. aeruginosa*, the types of diseases and prognosis vary (6). Previous studies have confirmed that there are differences in the specimen sources, affected populations, and *in vitro* antimicrobial susceptibility between mucoid and non-mucoid *P. aeruginosa*. In this study, we observed similar results that the rate of mucoid *P. aeruginosa* isolates among female patients (62.6%) exceeded that among male patients (37.4%). Conversely, non-mucoid *P. aeruginosa* was primarily isolated from male patients (61.7%). Regarding age distribution, it is notable that middle-aged women with chronic respiratory diseases appear particularly susceptible to mucoid *P. aeruginosa* infections. We also compared our results with the international research, which is a representative and comparative analysis of the demographic information of mucoid *P. aeruginosa* (3, 5, 13). The data from these international studies show a strong consistency with the results of this study. Both domestic and international studies indicate a predominance of female isolates in the gender composition (Fig. 3A). In terms of specimen types, the majority are derived from respiratory tract samples (Fig. 3B). Regarding the susceptibility of mucoid and non-mucoid isolates to main antimicrobial agents, while there is variability in resistance levels among isolates from different regions, the differences in resistance levels between mucoid and non-mucoid isolates are consistent, with these differences being more pronounced in Srifuengfung S’ and Owlia P’s studies. For instance, as illustrated in Fig. 3C (3) and Fig. 3D (5), the international data demonstrates that the resistance rates of non-mucoid *P. aeruginosa* are higher than those of mucoid isolates across all tested antimicrobial agents.

Fluoroquinolone, especially ciprofloxacin, plays a crucial role in the treatment of *P. aeruginosa* infections (15). However, our study demonstrates that fluoroquinolone might not be as effective in treating infections caused by mucoid *P. aeruginosa* as they are in treating non-mucoid isolates. Both types of *P. aeruginosa* exhibited the highest resistance rates to levofloxacin among the nine frequently used antimicrobial agents. When analyzing the therapy of *P. aeruginosa* infections, we found that the use frequency of levofloxacin for both phenotypes of *P. aeruginosa* was the highest among outpatients, which may contribute to the high rate of levofloxacin resistance. Ciprofloxacin was more susceptible than levofloxacin against mucoid and non-mucoid *P. aeruginosa*, which may explain the increased use of ciprofloxacin as a single agent or in combination after being reported. Ciprofloxacin was widely used in hospitalized patients with non-mucoid *P. aeruginosa* infections, either alone or in combination with other antimicrobial agents such as ceftazidime, meropenem, piperacillin/tazobactam, and colistin. However, its use in mucoid *P. aeruginosa* infections is limited. Piperacillin-tazobactam was the most effective antibiotic with a sensitivity rate of 89.7%, making it one of the cornerstone antimicrobial agents for treating *P. aeruginosa* infections (14). Several studies have found that macrolide antibiotics have the capability to inhibit the formation of biofilms and adhesion in mucoid *P. aeruginosa*. While they may not possess direct anti-*P. aeruginosa* activity, when used in conjunction with antibacterial agents that do have anti-*P. aeruginosa* activity, they can enhance the overall effectiveness of treatment (16–18).

The *in vitro* antimicrobial susceptibility testing of mucoid *P. aeruginosa* is challenging due to its slow growth rate. Therefore, the accuracy of such testing and its clinical guidance value for antimicrobial therapy remain unclear. This study evaluated the use of antimicrobial agents for the treatment of two different phenotypes of *P. aeruginosa* from two perspectives: first, a comprehensive analysis and comparison of all antimicrobial agents used based on the data from the pharmacy; second, considering that not all isolates may be true pathogens and the possibility of polymicrobial infections, we selected cases from hospitalized patients in which *P. aeruginosa* was the sole pathogen and reviewed the use of antimicrobial agents, treatment outcomes, and other relevant factors. The results revealed that no matter whether in mucoid or non-mucoid *P. aeruginosa*, the *in vitro* antimicrobial susceptibility test results were in good agreement with the therapeutic outcomes. Although mucoid *P. aeruginosa* is more likely to form biofilms, selecting appropriate antimicrobial agents based on *in vitro* susceptibility results still yields good therapeutic outcomes. In mucoid *P. aeruginosa*, the most frequently prescribed antimicrobial agents were levofloxacin and ceftazidime, while for non-mucoid *P. aeruginosa*, ceftazidime and meropenem were the most commonly prescribed. *In vitro* antimicrobial susceptibility results of *P. aeruginosa* are significant for the guidance of clinical antimicrobial agent selection, so it’s vital to consistently supervise the results.

We compared the medication use data from the pharmacy department and retrospectively reviewed cases. The case selection criteria included hospitalized individuals with clear infection indications and *P. aeruginosa* identified as the sole pathogen. This approach aimed to exclude the potential influence of mixed infections or colonization on the medication use data from the pharmacy department. The case results were generally consistent with the pharmacy department’s data, with minor discrepancies observed. Specifically, the data from the pharmacy department indicated that the most commonly used antimicrobial agents for mucoid *P. aeruginosa* patients were piperacillin-tazobactam, followed by ceftazidime. However, in the case review, ceftazidime was the most frequently used antimicrobial agent. This discrepancy may be attributed to the limited amount of case information available, leading to potential bias. Through the retrospective analysis of cases, our findings are not consistent with previous studies, which indicated contradictions between laboratory testing and clinical treatment outcomes (19).

This study represents a large-scale, single-center comparative research conducted over two years at a tertiary hospital in China. We found significant differences between mucoid and non-mucoid *P. aeruginosa* infections in terms of gender, antimicrobial susceptibility test, and the selection of antimicrobial agents. Specifically, mucoid *P. aeruginosa* infections were predominantly observed in females and were mainly associated with respiratory tract infections. Moreover, the isolates with mucoid phenotype exhibited higher susceptibility to most antimicrobial agents compared to non-mucoid isolates. However, antimicrobial susceptibility testing results effectively guided the selection of appropriate antimicrobial agents, leading to improved disease prognosis. Significantly distinct differences were observed in all these aspects, which have not been previously reported in existing research. We believe these findings will contribute to a comprehensive clinical understanding of the differences between mucoid and non-mucoid *P. aeruginosa*, guiding rational clinical management.

## SUPPLEMENTAL MATERIAL

Supplemental material spectrum.00287-25-s0001.docx Table S1; Fig. S1.

ASM does not own the copyrights to Supplemental Material that may be linked to, or accessed through, an article. The authors have granted ASM a non-exclusive, world-wide license to publish the Supplemental Material files. Please contact the corresponding author directly for reuse.
