# Supplementary material for: Comparison of in vitro antimicrobial susceptibility between mucoid and non-mucoid Pseudomonas aeruginosa and its guiding value for antibiotic therapy
Source: Microbiol Spectr. 2025 Jun 11;13(7):e00287-25. doi: 10.1128/spectrum.00287-25 (PMC12211038; doi:10.1128/spectrum.00287-25)

**Supplementary Table 1 Analyse the case information between mucoid and non-mucoid isolates.**

|  | Non-mucoid isolates (*n*=42) | Mucoid isolates (*n*=36) | *P* value |
| --- | --- | --- | --- |
| Average age* | 67 | 63 | 0.201 |
| Mortality^&^ | 6 (14.3%) | 3 (8.3%) | 0.441 |
| Unresolved^&^ | 5 | 0 | 0.034 |
| Average length of hospital stay* | 32d | 24d | 0.907 |
| Multiple agents use* | 33 (78.6%) | 20 (54.1%) | 0.105 |
| Agreement between in vitro  susceptibility testing and clinical outcomes* | 38 (90.2%) | 34 (94.4%) | 0.54 |

*The t test of two independent samples was used.

^&^The chi-square test was used.

**Supplementary Figure 1 Case Screening and Assessment.**


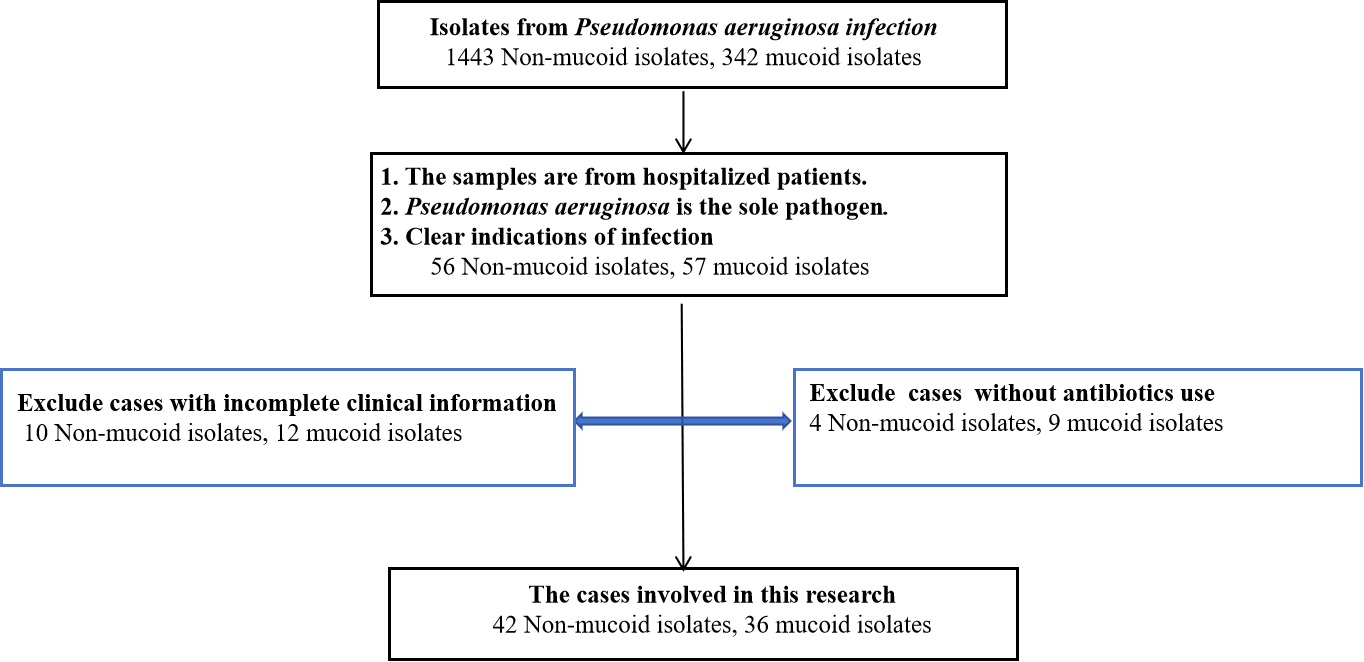

Supplement: Supplemental material — Table S1; Fig. S1. [file spectrum.00287-25-s0001.docx]
